# Prevalence of inherited retinal diseases in a large Egyptian cohort

**DOI:** 10.1186/s12886-023-03163-1

**Published:** 2023-10-20

**Authors:** Caroline Atef Tawfik, Maged Maher Roshdy, Nancy Magdy Morris

**Affiliations:** 1 Faculty of Medicine, Ain Shams University, 38 Abbasseya, Nour Mosque, El-Mohamady, Al Waili, 11566 Cairo, Egypt; 2 Watany Eye Hospital, Cairo, Egypt

**Keywords:** Autosomal recessive bestrophinopathy, Cone-rod dystrophy, Egypt, Inherited retinal diseases, Prevalence, Retinitis pigmentosa, Stargardt disease, Usher syndrome

## Abstract

**Background:**

Inherited retinal diseases form a rare, highly heterogeneous group of genetic disorders characterized by retinal degeneration. It is considered one of the leading causes of debilitating visual loss and blindness in children and young adults. Despite this few population-based data studies on prevalence of inherited retinal diseases exist. Moreover, prevalence can vary widely depending on geographical area, population ethnicity and cultural habits.

**Purpose:**

To report the prevalence of different subtypes of Inherited retinal diseases in a large Egyptian cohort in a retrospective, hospital-based, cross-sectional study.

**Methods:**

We conducted an extensive electronic medical record search for all the patients attending the outpatient clinic and investigation unit of Ain Shams University Hospital and the two branches of Watany Eye Hospital in the period between January 2015 and October 2022 aiming to identify the prevalence rate of different types of IRDs, patient demographics and stratify them according to their phenotype.

**Results:**

We examined the electronic medical records of 478 222 patients, 971 patients were diagnosed with IRD by clinical examination with or without any of the following investigations: color fundus photography, fundus autofluorescence, fundus fluorescein angiography, optical coherence tomography and/or electrophysiological studies as electroretinogram, visual evoked potential and electrooculogram. The overall prevalence was 0.2%. The most common IRD encountered was isolated retinitis pigmentosa with a percentage of 78.9% followed by Stargardt disease at 6.3%, cone-rod dystrophy at 2.0%, autosomal recessive bestrophinopathy at 1.9% and unspecified IRD at 1.5%.

**Conclusion:**

Retinitis pigmentosa was the most common IRD encountered followed by Stargardt disease. Many of the dystrophies are the subject of clinical intervention trials, and population-based epidemiological data can guide phenotype-based genetic testing and help assess the future need for treatment.

## Background

Inherited retinal diseases (IRDs) form a rare, highly heterogeneous group of genetic disorders characterized by retinal degeneration. Many subtypes exist where the classification is usually difficult, complex and variable according to the mode of inheritance, age of onset, disease progression rate, the primary site of retinal dysfunction (photoreceptors either rods, cones or both, retinal pigment epithelium, or inner retina and choroid) and whether they are associated with syndromic features or not [1].

IRDs is considered one of the leading causes of debilitating visual loss and blindness in children and young adults between the age of 15 and 45 years [2], with more than one and a half million affected individuals worldwide [3].

Many dedicated studies have reported the prevalence rate of each subtype of IRDs which can be usually found in the Online Mendelian Inheritance in Man (OMIM) database (http://www.ncbi.nlm.nih.gov/omim). However, few population-based data studies on the prevalence of IRDs exist. Moreover, prevalence can vary widely depending on geographical area, population ethnicity (whether ethnically heterogeneous or homogeneous) and cultural habits [4–5]. In fact, retinitis pigmentosa (RP) prevalence, often reported as 1/4000 in developed countries, can reach up to 1/230 in populations with high consanguinity rates [6, 7] as is the case among Egyptians.

There is a scarcity of comprehensive reports of IRDs within the Egyptian population and the knowledge of the IRD prevalence in Egypt remains limited. Moreover, despite being in the era of next-generation sequencing (NGS), genetic testing of IRD genes is, for the most part, not covered by health insurance, so the tests are usually not offered by health care providers.

The purpose of this study was to characterize current clinical knowledge of patients with IRDs in Egypt and estimate their prevalence, thereby providing a foundation for the assessment of current and future healthcare needs for these patients, serving as a basis for devising cost-effective population-specific genetic tests and assuring early treatment with new therapies.

## Methods

A retrospective, hospital-based study was conducted at Ain Shams University as well as both branches of a high-volume ophthalmology hospital with a catchment area from all over Egypt; Watany Eye Hospital, Cairo, Egypt. We conducted an extensive electronic medical record search for all the patients attending the outpatient clinic and investigation unit in the period between January 2015 and October 2022 aiming to identify the prevalence rate of different subtypes of IRDs, study the patient demographics, and stratify them according to their phenotype.

We extracted the cases from free text by applying the ‘text mining technique’ to improve case detection as well as minimize missing cases and bias findings. We used various keywords (as a whole term and as each word alone) including (but not limited to): dystrophy, inherited, consanguinity, familial, recessive, dominant, X-linked, night blindness, retinitis pigmentosa, Usher syndrome, Bardet-Biedl, Leber congenital amaurosis, retinoschisis, Stargardt, bestrophinopathy, ARB, Best, pattern, FEVR, occult, macular dystrophy, North Carolina macular dystrophy, enhanced S-cone syndrome, achromatopsia, cone dystrophy, rod-cone dystrophy, cone-rod dystrophy, Joubert syndrome, Senior-Løken syndrome, Alström, Refsum, etc. The entries we extracted were further scrutinized by C.A.T. (an IRD expert) to verify the consistency of data as well as review their investigations if any. Any inconsistent set of data or wrong cases were excluded.

The patients were diagnosed with IRD either by clinical examination with or without one or more of the following investigations: color fundus photography, fundus autofluorescence (FAF) and/or fundus fluorescein angiography (FFA), spectral-domain optical coherence tomography (SD-OCT), visual field perimetry (VF) and electrophysiological studies as electroretinogram (ERG), visual evoked potential (VEP) and electrooculogram (EOG) recorded according to the International Society for Clinical Electrophysiology (ISCEV) standards.

For the diagnosis of RP, we relied upon the criteria proposed by Bertelsen et al. in 2014 excluding the presence of pathogenic mutation as most of our patients didn’t undergo genetic testing (Table 1) [8]. Moreover, the diagnosis of Usher syndrome was based on presence of RP phenotype associated with hearing difficulty (early onset, bilateral and not otherwise explained by another disease) and/or balance problems. Lastly, the diagnosis of Bardet-Biedl syndrome (BBS) was based on the modified diagnostic criteria proposed by Beales et al. in 1999 (Table 2) [9].

**Table 1:** Diagnostic criteria for retinitis pigmentosa (RP) [8]

Diagnosis is based on Either: Night blindness + ophthalmoscopic evidence or Electroretinogram (ERG), OR Ophthalmoscopic evidence + Visual field (VF) or ERG evidence, OR VF defects + ERG	
Night blindness	Anamnestic or demonstrated by dark adaptometry
Ophthalmoscopic evidence	Narrow retinal arterioles, diffuse and widespread atrophy of the retinal pigment epithelium and/or choroid, bony spicules or granular hyperpigmentation, abnormality of fundus reflexes and/or optic nerve head pallor
Visual field defects	Peripheral or mid-peripheral
Dark-adapted electroretinogram (ERG)	Severe reduction or extinction

**Table 2:** Modified diagnostic criteria for Bardet-Biedl Syndrome [9]

Diagnosis is based on four primary criteria OR three primary plus two secondary criteria
*Primary Criteria*
Rod-cone dystrophy Polydactyly Obesity Learning disabilities Hypogonadism in males Renal anomalies
*Secondary Criteria*
Speech disorder/delay Strabismus/cataracts/astigmatism Brachydactyly/syndactyly Developmental delay Polyuria/polydipsia Ataxia/poor coordination/imbalance Mild spasticity (especially lower limbs) Diabetes mellitus Dental crowding/hypodontia/small roots/high arched palate Left ventricular hypertrophy/congenital heart disease Hepatic fibrosis

For the sake of unification of the different phenotypes we encountered in our study, we grouped them into 13 clinical categories. For each patient, the variables studied included residence, sex, age at presentation, family history, refractive error, investigations he/she underwent if any, and lens status. The best-corrected visual acuity (BCVA) was recorded using a Snellen chart and converted to the logarithm of the minimum angle of resolution (LogMAR) using a validated procedure [10].

### Statistical analysis

The statistical analysis was performed using MedCalc Statistical Software version 20.115 (MedCalc Software Ltd, Ostend, Belgium). Means, standard deviations (SDs), Odds ratios (OR), and 95% confidence intervals (CI) were calculated. A p-value of < 0.05, measured by Welch’s test, indicated statistical significance.

## Results

We examined the electronic medical records of patients attending the outpatient clinics and investigations unit. The records of 478 222 patients coming from all the 27 Egyptian governorates were included, out of which 971 patients were diagnosed with IRDs based on clinical examination and/or investigations or a combination of both. The median age was 35 years (IRQ 24 to 49) and ranged from 1.5 to 98 years, by stratifying the patients according to decades of age, the most prevalent age group was in the fourth decade of age accounting for 24.3% of the patients. Males accounted for 59.5% (578) of patients diagnosed with IRDs (Table 3).

**Table 3:** The phenotypes encountered in the study were grouped into 13 clinical diagnosis categories along with percentage of patients in each category and sex distribution

Clinical Diagnosis Category	Phenotypes included in categories	n = (% of total)	Men
Bardet-Biedl Syndrome	Bardet-Biedl syndrome	11 (1.10%)	7
Bestrophinopathies	Best’s vitelliform macular dystrophy, autosomal recessive bestrophinopathy, Adult-onset vitelliform macular dystrophy	27 (2.80%)	10
Cone dystrophy	Cone dystrophy	2 (0.20%)	2
Cone-Rod dystrophy	Cone-rod dystrophy, Enhanced S-cone syndrome	25 (2.60%)	17
Congenital stationary night blindness	Congenital stationary night blindness (complete or incomplete), fundus albipunctatus	2 (0.20%)	2
Chorioretinal dystrophy	Central areolar choroidal dystrophy, choroideremia, gyrate atrophy, pigmented paravenous chorioretinal dystrophy, Bietti’s crystalline retinopathy, late-onset retinal dystrophy	10 (1.00%)	3
Leber’s Congenital Amaurosis	Leber’s Congenital Amaurosis	10 (1.00%)	6
Macular dystrophy, other	Occult macular dystrophy	2 (0.20%)	1
Retinitis pigmentosa, isolated	Retinitis pigmentosa (all inheritance forms), retinitis punctata albescens, pericentral and sector retinitis pigmentosa	769 (79.20%)	459
Retinitis pigmentosa, syndromic	Joubert syndrome, neuronal ceroid lipofuscinosis, Refsum syndrome	5 (0.50%)	4
Stargardt disease	Stargardt disease, flecked retina	62 (6.40%)	36
Usher’s syndrome	Usher’s syndrome (all types)	23 (2.40%)	12
X-linked retinoschisis	X-linked retinoschisis	8 (0.80%)	8

Fifty-seven patients (5.9%) came from consanguineous families, while no data was available in 58.3% of cases. Positive family history (at least one family member affected by the disease) accounted for 3.6% of cases and no available data was present in 3.3%.

Reviewing the lens status of the IRD patients, cataract was found in 39.2% (n = 380) of patients, out of which 48.4% (n = 184) underwent cataract surgery.

Regarding the refractive error, following the exclusion of aphakic cases, the mean error was − 0.75 D (SD 4.25 D) (median − 0.25 D, range − 19.75 to + 25 D). On the other hand, the mean BCVA measured in LogMAR was 1.27 (median 1, range 0 to 4) (SD 1.16).

**Fig. 1:**
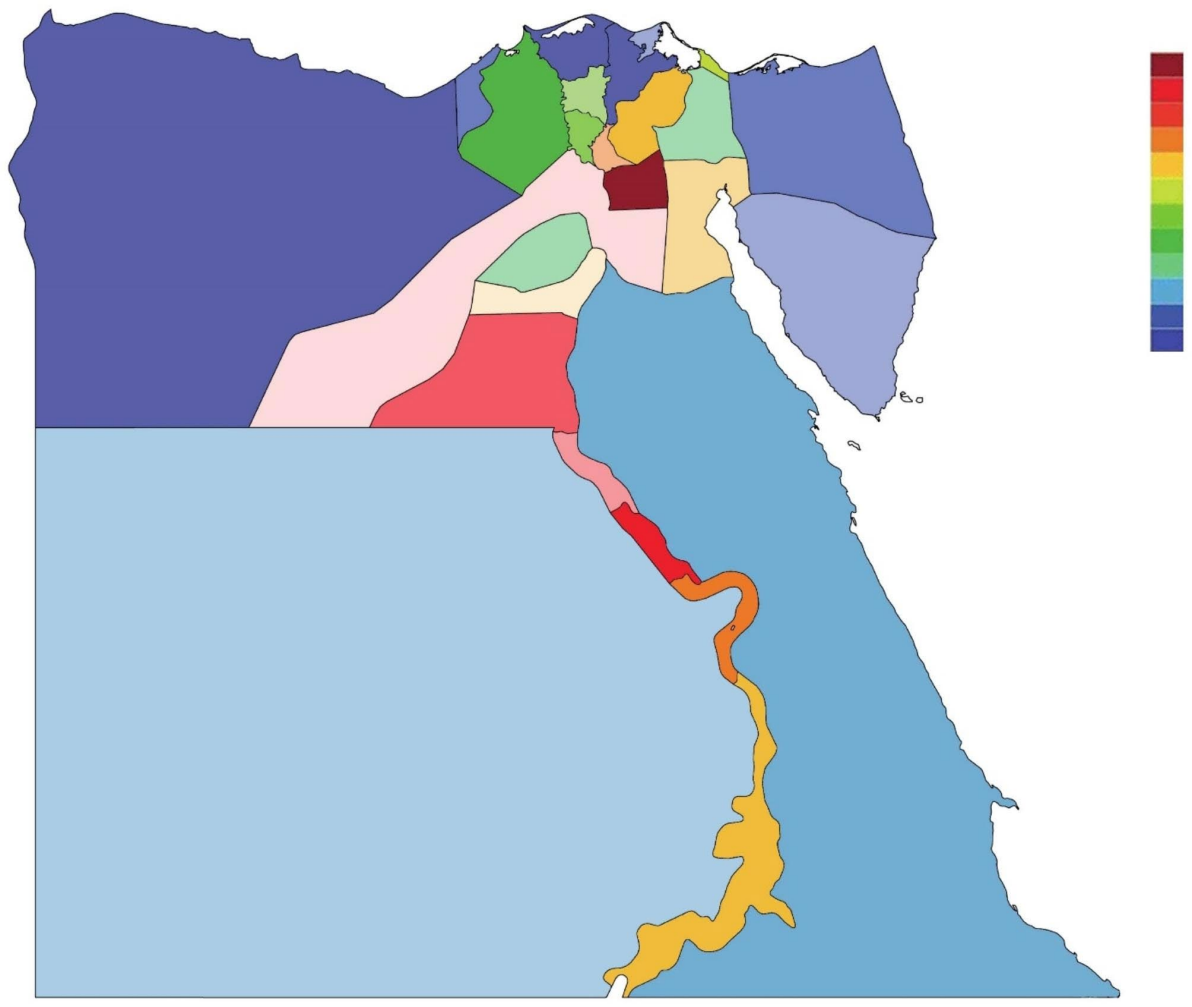
Map of Egypt with its governorates showing the geographical distribution of our cohort in a color-coded manner [color spectrum ranges from cool to warm colors, where warmer colors (orange/red) indicate higher values, while cooler colors (indigo/blue) indicate lower ones]

Regarding the geographical distribution of the IRD patients, Cairo governorate had the leading number of patients (n = 427) representing 44% of the cohort. Sohag governorate followed by 80 patients (8.2%), then Minya governorate with 78 (8.0%), and Asyut governorate with 69 (7.1%) (Fig. 1).

**Fig. 2:**
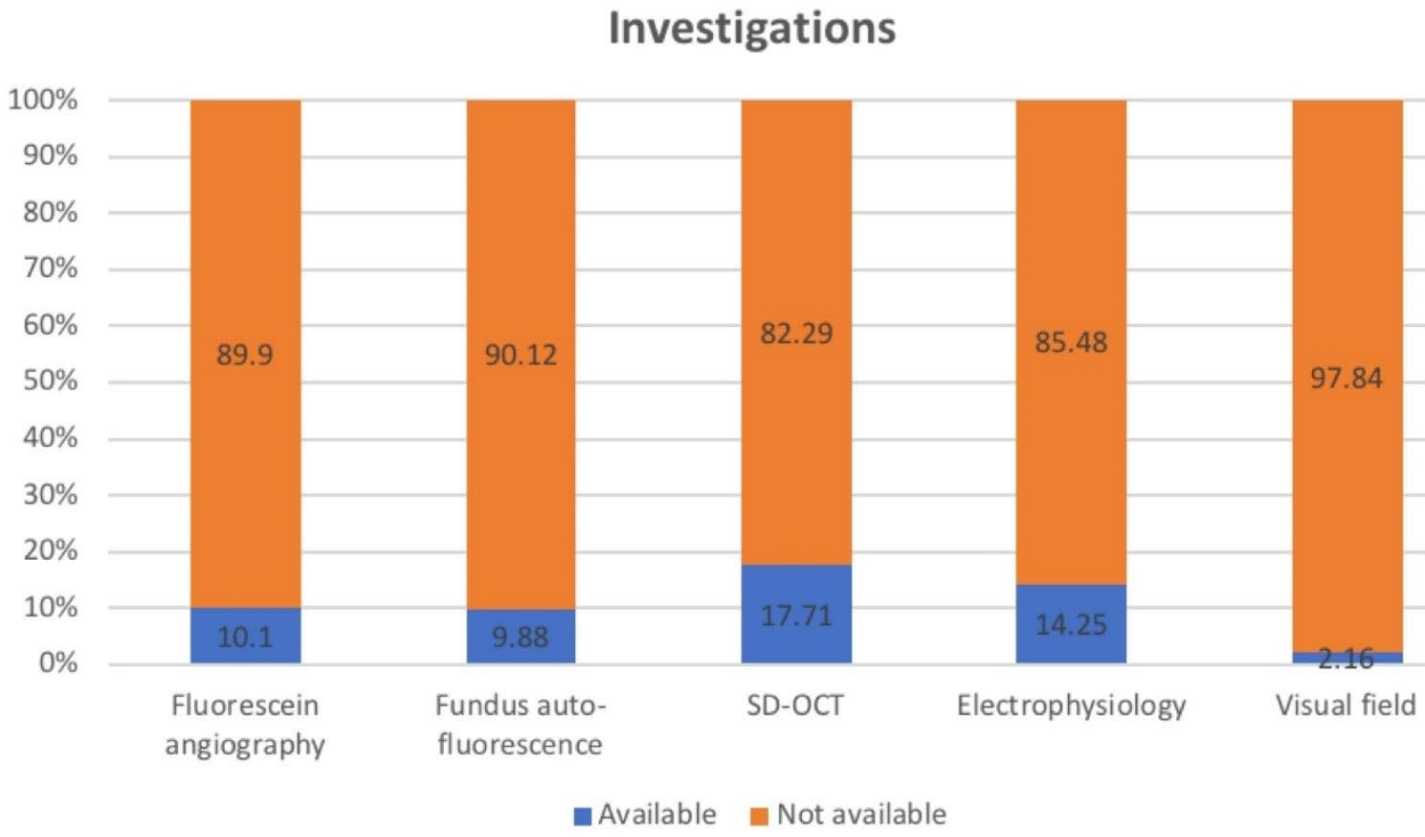
Bar chart showing the percentage of available investigations for our cohort. SD-OCT; spectral-domain optical coherence tomography

The overall prevalence of IRDs in our cohort was 0.2% (1:500). The diagnosis was based solely on clinical examination in 70.2% (n = 682), and on both clinical and investigations (imaging and/or functional testing) in 29.8% where SD-OCT was the most common investigation ordered accounting for 17.7% (n = 172) followed by ERG by 14.5% (n = 141) while 10.1% underwent FAF and/or FFA and only 2.2% underwent VF (Fig. 2).

The most common IRD phenotype encountered was isolated, non-syndromic RP accounting for 78.9% (n = 766), followed by Stargardt disease (STGD) phenotype with 6.3% (n = 61), Usher syndrome with 2.4% (n = 23), Cone-rod dystrophy (CRD) with 2.0% (n = 19) and autosomal recessive bestrophinopathy (ARB) accounting for 1.9% (n = 18).

## Discussion

A population-based analysis and estimated prevalence of IRDs in Egypt has, to our knowledge, not been reported before. Egypt is still lagging behind in terms of diagnostics and the application of new techniques as the public healthcare system is largely underfunded.

IRDs are rare disease entities where an average general ophthalmologist can see only a few cases during his or her entire career, and this contributes to the difficulty in reaching the correct clinical diagnosis which could direct the genetic testing to be as focused and least expensive as possible. A gap of knowledge exists regarding the prevalence of this group of blinding diseases in Egypt which will be amenable to therapy in the near future.

It is quite challenging to determine the IRDs diagnoses, especially without genetic testing, mainly due to clinical heterogeneity and considerable overlap of phenotypes among the different types of IRDs. These factors explain the large number of undiagnosed cases as well as the lack of conclusive clinical diagnosis in other cases.

Other factors that can hinder definitive diagnoses are lack of information about symptoms and signs of different disease entities, lack of accurate family history and proper pedigree labelling, time of patient evaluation whether early or late in the natural course of the disease, the presence of confounding characteristics, and difficulty of disease progression monitoring. Moreover, the lack of awareness among general ophthalmologists of IRDs as well as the scarcity of IRD experts with special training who can recognize the different subtypes of IRDs, contribute to the missing or delay in identifying the phenotype which is crucial to focus genetic testing.

The overall prevalence of IRDs in our study was 0.2% i.e. 1:500. This was much higher than reported rates from Norway (1:3856), [11]. Denmark (1:3454), [8] and Northern France (1:1490) [12]. Regarding the clinical spectrum, the most common IRD encountered was isolated RP with a prevalence of 0.16% (1:625) confirming as in other studies that it is the most prevalent of all IRDs. This is comparable to the prevalence in South India (1:570) [5] and Beijing Eye Study (1:1000) [7] but lower than that reported in Puerto Rico (1:229) [13]. However, lower prevalence rates were detected in the United States of America (1:3700), [14]. Norway (1:4440), [15]. Birmingham city in the United Kingdom (1:4869), [16]. Slovenia (1:6023), [17] and Switzerland (1:7000) [18]. This could be attributed to less accurate estimation owing to a relatively small studied population as well as diagnoses being largely based only on clinical examination or fundus appearance. Moreover, consanguinity rates vary from one population to another with variable religious, cultural, and geographical factors (urban/rural community, size of area, and population isolation) [19]. High rates of consanguinity are observed in most communities of North Africa and the Middle East as well as South India with high rates of inbreeding accounting for 20–50% of all marriages [20, 21]. Egypt is no stranger to consanguinity, where it is deeply rooted in some communities mainly in Upper Egypt and rural areas. Lastly, the higher prevalence may be related to the fact that the hospitals are tertiary care hospitals with cases referred from all over Egypt.

Among IRDs, the most common syndromic IRD encountered in our study was Usher syndrome accounting for 2.4% of cases, thereby agreeing with previous reports from South Africa [22]. Brazil [23], and Northern France [12]. BBS, the second most common syndromic RP, accounted only for 1.1% of cases, which corresponds roughly to half of those with Usher syndrome. A plausible explanation is the fact that the nine known genes responsible for Usher syndrome are very large genes with a greater probability of mutation occurrence than in diseases caused by smaller genes [24].

As for macular dystrophies, STGD was the most frequently encountered disease entity in our study comprising 6.3% of the cases, this was aligned with most of the other studies [25]. There was a wide range of prevalence rates reported ranging from 5.51% in Southern France [24] up to 20.62% in Brazil [23]. This wide variability could be attributed to the difficulty in ensuring a definite diagnosis based on fundus appearance, and the geographical variability related to underlying gene mutations as seen in North Carolina macular dystrophy (NCMD).

Lastly, 1.5% of cases encountered in our study couldn’t be accurately diagnosed and remain unclassified, this highlights the importance of raising awareness among ophthalmologists about IRDs and the multi-modal imaging as well as functional studies that may be needed to reach a probable diagnosis. Moreover, many cases require referral to an IRD expert who can proceed to do deep phenotyping to reach the clinical diagnosis, which can guide further genetic testing.

As it was expected that the IRD diagnosis will be missed in many patients, the reported prevalence (0.2%) of all IRDs is the minimum prevalence. Likewise, RP is reported to be the most common not just for being so, but also due to its characteristic fundus appearance and striking presenting symptom of night blindness that usually pushes the patient to seek medical advice.

Regarding age, the median age of patients was 35 years which agreed with that reported in Norway (42.7 years), [11]. Korea (44.8 years) [26] and Australia (46 years) [27] yet was less than that reported in South India (53.9) [5] and Beijing eye study (57 years) [7], thus reflecting relative diagnostic delay. The delay in seeking medical advice in children and young adults is not uncommon and may result from the deferment of patients with referring symptoms and/or lack of awareness and expertise in IRDs among ophthalmologists. This is commonplace with any rare disease as per the data from the Survey of the Delay in Diagnosis for Rare Disease in Europe [28]. Moreover, children with unappreciated disabilities who hardly complain along with minimal fundus changes can contribute to this delay significantly.

Our study showed preponderance for males accounting for 59.5%, this could be contributed to men having more access to healthcare services as well as the inclusion of X-linked diseases within the cohort.

Cataract was found in 39.2% of our cohort with 35.3% associated with isolated RP and 1.3% with Usher syndrome, thereby confirming that cataract is an important co-morbidity in RP whether isolated or syndromic and causes a significant impairment of central visual function to patients whose peripheral visual functions are already compromised. The prevalence of cataract found in our study agrees with those reported by Pruett (46.5%) [29] and Fishman et al. (41%) [30]. These findings highlight the importance of monitoring IRD patients for the occurrence of cataract and their proper timely management.

To the best of our knowledge, our study is the first of its kind among the Egyptian population, we were able to include a wide age range of patients coming from different regions of the same country. However, our study had limitations, most notably the discrepancy follow-up between different patients where some had only one visit, and others several ones over a few years. Moreover, the age of diagnosis was elusive, as we couldn’t ensure when exactly the patient received the diagnosis. Some remained undiagnosed till we could review their records, further emphasizing the importance of review by an IRD expert. Lastly, the hospital-based study design may affect the overall population frequency as it limits the data to those only whose data were included in the given hospitals.

## Conclusion

Many of the IRDs are the subject of clinical intervention trials, and population-based epidemiological data can guide phenotype-based genetic testing and help assess the future need for treatment.

Ongoing clinical intervention trials show a lot of progress. Nowadays, with the availability of gene augmentation therapy for biallelic *RPE65* disease, more hope is on the horizon. More therapeutic options will be made available within the next few years, to patients with IRDs for whom the only current remedy existing is rehabilitation. The population-based prevalence and diagnostic spectrum may be used to estimate the burden of these diseases and for proper planning of phenotype-guided genetic testing and future need for treatment.

## Data Availability

All data generated or analyzed during this study are included in this article. Further enquiries can be directed to the corresponding author.

## Abbreviations

IRD: Inherited retinal disease
RP: Retinitis pigmentosa
NGS: Next generation sequencing
FAF: Fundus autofluorescence
FFA: Fundus fluorescein angiography
SD-OCT: Spectral-domain optical coherence tomography
VF: Visual field perimetry
ERG: Electroretinogram
VEP: Visual evoked potential
EOG: Electrooculogram
ISCEV: International society of clinical electrophysiology of vision
BBS: Bardet-Biedl syndrome
BCVA: Best corrected visual acuity
LogMAR: Logarithm of the minimum angle of resolution
SD: Standard deviation
OR: Odds ratio
CI: Confidence interval
IQR: Interquartile range
STGD: Stargardt disease
CRD: Cone-rod dystrophy
ARB: Autosomal recessive bestrophinopathy
NCMD: North Carolina macular dystrophy
